# Identification and targeted management of a neurodegenerative disorder caused by biallelic mutations in SLC5A6

**DOI:** 10.1038/s41525-019-0103-x

**Published:** 2019-11-14

**Authors:** Alicia B. Byrne, Peer Arts, Steven W. Polyak, Jinghua Feng, Andreas W. Schreiber, Karin S. Kassahn, Christopher N. Hahn, Dylan A. Mordaunt, Janice M. Fletcher, Jillian Lipsett, Drago Bratkovic, Grant W. Booker, Nicholas J. Smith, Hamish S. Scott

**Affiliations:** 1 Genetics and Molecular Pathology Research Laboratory, Centre for Cancer Biology, An alliance between SA Pathology and the University of South Australia, Adelaide, SA Australia; 2 School of Pharmacy and Medical Sciences, University of South Australia, Adelaide, Australia; 3 School of Biological Sciences, University of Adelaide, Adelaide, SA Australia; 4 ACRF Cancer Genomics Facility, Centre for Cancer Biology, An alliance between SA Pathology and the University of South Australia, Adelaide, SA Australia; 5 Department of Genetics and Molecular Pathology, SA Pathology, Adelaide, SA Australia; 6 School of Medicine, University of Adelaide, Adelaide, SA Australia; 7 South Australian Clinical Genetics Service, Women’s and Children’s Hospital, North Adelaide, SA Australia; 8 Department of Surgical Pathology, SA Pathology, North Adelaide, SA Australia; 9 Department of Neurology, Women’s and Children’s Hospital, North Adelaide, SA Australia

**Keywords:** Medical genomics, Neurodevelopmental disorders, Personalized medicine, Translational research, Metabolic disorders

## Abstract

We describe a sibling pair displaying an early infantile-onset, progressive neurodegenerative phenotype, with symptoms of developmental delay and epileptic encephalopathy developing from 12 to 14 months of age. Using whole exome sequencing, compound heterozygous variants were identified in *SLC5A6*, which encodes the sodium-dependent multivitamin transporter (SMVT) protein. SMVT is an important transporter of the B-group vitamins biotin, pantothenate, and lipoate. The protein is ubiquitously expressed and has major roles in vitamin uptake in the digestive system, as well as transport of these vitamins across the blood–brain barrier. Pathogenicity of the identified variants was demonstrated by impaired biotin uptake of mutant SMVT. Identification of this vitamin transporter as the genetic basis of this disorder guided targeted therapeutic intervention, resulting clinically in improvement of the patient’s neurocognitive and neuromotor function. This is the second report of biallelic mutations in *SLC5A6* leading to a neurodegenerative disorder due to impaired biotin, pantothenate and lipoate uptake. The genetic and phenotypic overlap of these cases confirms mutations in *SLC5A6* as the genetic cause of this disease phenotype. Recognition of the genetic disorder caused by *SLC5A6* mutations is essential for early diagnosis and to facilitate timely intervention by triple vitamin (biotin, pantothenate, and lipoate) replacement therapy.

## Introduction

While individually rare, inborn errors of metabolism are over-represented amongst the childhood neurodegenerative diseases, with an increasing number of novel disorders identified.^1^ These disorders are caused by perturbed transport or processing of essential metabolites and have a profound impact on nervous system development and function. In the majority of cases, this effect is compounded by a lack of effective therapy. However, contemporary elucidation of the molecular abnormalities underpinning these disorders is informing therapeutic development, with early intervention translating to favourable clinical responses in a growing number of cases.^2^

Highlighting the importance of recognising novel, ultra-rare disorders with the potential for personalised therapeutic intervention is the recently described disorder of sodium-dependent multivitamin transport (SMVT).^3,4^ The SMVT protein (encoded by *SLC5A6*) is ubiquitously expressed and plays a major role in the uptake of biotin, pantothenate and lipoate in the digestive system, as well as in transporting these B-group vitamins across the blood–brain barrier. This report showed that pathogenic mutations in *SLC5A6* resulted in aberrant cellular uptake of these critical organic cofactors, manifesting clinically in a single patient as a multisystem disease with infantile neurocognitive regression.^4^

Here we describe a second, non-consanguineous sibling pair, presenting in early infancy with a progressive neurodegenerative phenotype. Whole exome sequencing (WES) revealed compound heterozygous mutations in *SLC5A6*, with pathogenicity of the identified variants demonstrated by impaired biotin uptake of mutant SMVT. Targeted vitamin replacement therapy was initiated resulting in sustained clinical improvement, providing further evidentiary support for disease causality and expanding the recognised spectrum of inborn diseases of biotin metabolism.^4–8^

## Results

### Family presentation

**Fig. 1:**
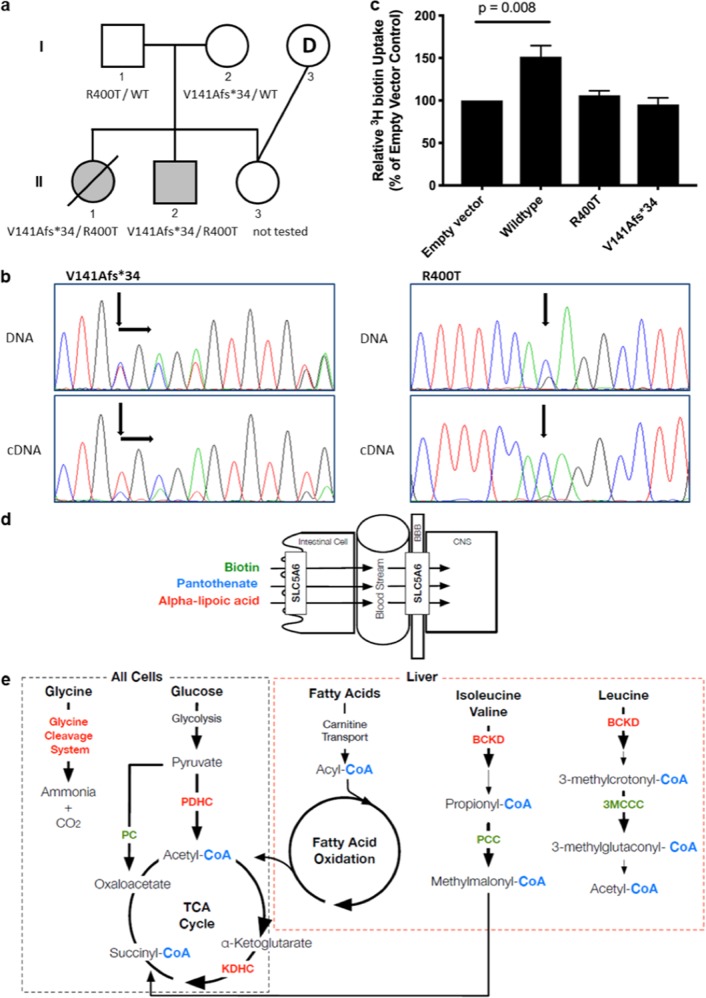
Biotin, pantothenate and lipoate-dependent metabolic pathways and the effect of identified variants in SLC5A6. **a** Pedigree of the non-consanguineous family. **b** Chromatograms from Sanger sequencing of patient DNA compared to cDNA show decreased expression of the V141Afs*34 allele and stable expression of the R400T allele. **c** Uptake of radiolabelled biotin by HeLa cells transfected with empty vector, wild-type or mutant SMVT expression constructs. Uptake by mutant constructs is decreased compared to wild-type (*p* = 0.008) and not significantly different to empty vector (*p* > 0.05). Data show the mean and standard error of the mean (*n* = 4). **d** SCL5A6 function and **e** Enzymes for which the vitamins Biotin (green), Pantothenate (blue) and Alpha-lipoic acid (red) play a role as important cofactors in: the degradation pathways of the amino acids leucine, isoleucine, valine and glycine; glucose energy metabolism; the TCA cycle; and fatty acid oxidation metabolism. All pathways apart from the glycine cleavage system play a fundamental role in cellular energy production. The pathways involved with fatty acid metabolism and branch chain amino acid breakdown occur almost exclusively in the liver. BBB blood–brain barrier, BCKD Branch chain ketoacid dehydrogenase, CNS central nervous system, KDHC ketoglutarate dehydrogenase complex, PC pyruvate carboxylase, PCC propionyl-CoA carboxylase, PDHC pyruvate dehdrogenase complex, 3MCCC 3-methyl crotonyl-CoA carboxylase

Two siblings (II-1 and II-2) presenting with a profound neurodevelopmental delay during infancy were born to unaffected, unrelated parents (I-1 and I-2). Before a molecular diagnosis was determined for II-1 and II-2, the parents chose an ovum donor (I-3) to reduce the risk of recurrence in their third child (II-3) (Fig. 1a).

Detailed clinical descriptions of both affected siblings can be found in the supplementary information and are summarised in Table 1. In brief, both siblings were born at term without complication following a benign antenatal period. Birth occipitofrontal head circumference (OFC) was at the 50th centile with 34 cm (II-1) and 34.5 cm (II-2), followed by postnatal reduction (2nd–10th centile) in both children. Early developmental progress was age appropriate with a plateau in global development from 14 months (II-1) and 12 months (II-2). II-1 walked independently (15 months), however regressed to crawling (20 months) and non-ambulation (24 months). Her vocabulary of 15–20 words (15 months) retracted to 10 words (23 months), only using ‘mum’ and ‘dad’ by 30 months. II-2 commenced few independent steps at 12 months and used a walking assist device until 5 years and 6 months when he regressed to crawling. His vocabulary of 10 single words by 15 months regressed to non-verbal by 24 months.

**Table 1:** Clinical features and outcome following treatment of the three patients with biallelic SLC5A6 mutations causing SMVT deficiency

Clinical features	This study—patient II-1	This study—patient II-2	Subramanian 2017
***Neurological***			
Neurocognitive regression	Onset: 14 months	Onset: 12 months *Post treatment*: Neurocognitive progress^a^ -walking with assistance -4–6 word vocabulary -improved social interaction/attention -utilising cup and spoon	Onset: infantile *Post treatment*: Neurocognitive progress^b^ -walking with assistance -5-word vocabulary -improved social interaction/personality
Microcephaly	Yes, relative^c^	Yes, relative^c^	Yes
Neuro-ophthalmological	Left esotropia	Nystagmus Dyskinetic saccades Binocular esotropia *Post treatment*: Nystagmus marked reduction	Nystagmus *Post treatment*: Nystagmus resolved
Spasticity	No	No	Yes
Hyperreflexia	Yes	Yes^d^	NR
Seizures	No	Yes (well controlled) *Post treatment*: No significant change Reduced anticonvulsant requirement	NR
Hyperacusis	Yes	Yes (resolved pre-treatment)	NR
Peripheral neuropathy	NR	Mixed demyelinating & axonal sensorimotor polyneuropathy *Post treatment*: Electrographically resolved^e^	NR
Neuroimaging (MRI)^f^	No cerebral atrophy Right cerebellar haemorrhagic foci T2/FLAIR signal hyperintensity (periventricular & parieto-occipital white matter)	Cerebral atrophy (progressive) Cerebellar atrophy (progressive) Brainstem (pontine) atrophy Thin corpus callosum T2/FLAIR signal hyperintensity (central segmental tract & peritrigonal regions) Mega cisterna magna 1H-MRS (SVS) [31 and 144 ms; basal ganglia/frontoparietal white matter]: reduced NAA and broad lactate doublets	Cerebral atrophy Brainstem (pontine) atrophy Thin corpus callosum
Electroencephalogram (EEG)^g^	Not done	Background slowing (encephalopathy) Epileptiform activity: generalised and multifocal spike-wave (2–3 Hz) *Post treatment*: Improved background rhythms^h^ Epileptiform activity reduced	Normal
Histopathology	Central nervous system: axonal spheroids Peripheral nervous system: undefined thickening Skeletal muscle biopsy: denervation atrophy	Cutaneous biopsy: membranous cytoplasmic inclusions^i^	Skeletal muscle biopsy: normal
***Gastrointestinal***			
Feeding difficulties/ failure to thrive	Yes, bulbar dysfunction	Yes, bulbar dysfunction	Yes
Nasogastric tube/ gastrostomy feeding^j^	Yes	Yes	Yes
GI haemorrhage	Yes^k^	Yes^l^	Yes^m^
Other	GORD	Cyclical vomiting GORD *Post treatment*: cyclical vomiting improved	NR
***Cardiopulmonary***			
	Asthma Right heart failure^n^	Asthma tracheobronchomalacia ECG: non-specific ST & T-wave changes^o^	NR
***Other***			
Hypogammaglobulinemia	NR	Yes, Isolated IgG deficiency *Post treatment*: NR	Yes, IgG/IgA deficiency *Post treatment*: Resolved
Osteopenia	No	No	Yes *Post treatment*: Resolved
Digital clubbing	NR	Yes^p^	NR
***Treatment***			
	Nil	Biotin (intramuscular) 10 mg weekly Dexpanthenol (intramuscular) 250 mg weekly α-lipoic acid (intravenous) 300 mg weekly	Biotin (oral) 10–30 mg/day Pantothenic acid (oral) 250–500 mg/day α-Lipoic acid (oral) 150–300 mg/day

*GORD* gastro-oesophageal reflux disease, NR not reported

^a^Reported at +5 years from commencement of therapy

^b^Reported at +2 years, 9 months from commencement of therapy

^c^Relative postnatal microcephaly (OFC growth retardation)

^d^Initial hyperreflexia; progressive obtundation of myotatic reflexes presumed secondary to peripheral neuropathy

^e^Confirmed by serial nerve conduction studies pre/post treatment

^f^Most recent pre-treatment study [age]: II-1 [1 year, 12 months]; Supplementary Fig. 2A, II-2 [7 years, 5 months]; Supplementary Fig. 2B and C, Subramanian [12 months]

^g^Most recent pre-treatment study [age]: II-2 [5 years, 5 months], Subramanian [6 months]

^h^Post-treatment EEG [+4 years, 8 months]

^i^Supplementary Fig. 3B

^j^Insertion: II-1 (nasogastric tube [2 years, 7 months]; nil gastrostomy), II-2 (gastrostomy [6 years, 3 months]), Subramanian (nasogastric tube [infancy]; gastrostomy [6 months])

^k^Perforated duodenal ulcer/duodenal artery

^l^Suspected Mallory–Weiss tear in context of cyclical vomiting

^m^Reported secondary to thrombocytopenia

^n^Features of right heart failure at autopsy, presumed secondary to pre-mortem cardiopulmonary resuscitation

^o^No clinical cardiomegaly and normal screening echocardiogram [8 years, 9 months]; non-specific ST segment and T wave changes present on ECG

^p^Digital clubbing of uncertain aetiology (note: normal high-resolution chest CT)

Neurological assessment demonstrated progressive truncal ataxia with dyskinetic appendicular movements from 14 months (II-1) and 12 months (II-2). II-2 developed mixed semiology seizures including focal dyscognitive, absence, tonic spasms and generalised convulsive seizures with electrographic features of encephalopathy with generalised and independent multifocal spike-wave discharges (Supplementary Fig. 1A). The seizures proved well controlled on a regimen of levetiracetam, topiramate and vigabatrin. Refractory cyclical vomiting (II-2) was present from 3 years, 5 months of age. As neurophysiological assessment, neuroimaging, biochemical, single-gene testing and mitochondrial genome sequencing did not provide a diagnosis for this family, WES was performed.

At 2 years 7 months, II-1 died secondary to acute gastrointestinal haemorrhage following perforation of a duodenal ulcer. Post-mortem examination revealed a small cerebrum and cerebellum for age (brain weight 977 g), with preservation of overall neural architecture. Cardiomegaly, hepatic congestion and aspiration pneumonia were also observed, likely secondary to pre-mortem cardiopulmonary resuscitation. Histopathological analysis demonstrated a global increase in glial density with reactive gliotic change. Axonal spheroids, up to 100 μm in diameter, were ubiquitously present (Supplementary Fig. 3A). Peripheral nerves demonstrated variable axonal irregularity and patchy denervation atrophy was evident within skeletal muscle.

### Treatment outcomes

Upon identification of the pathogenic *SLC5A6* mutations, II-2 commenced parenterally administered triple vitamin replacement therapy at 7 years and 1 month of age, with doses of Biotin (10 mg, intramuscular), Dexpanthenol (250 mg, intramuscular) and α-lipoic acid (300 mg, intravenous) given weekly. To date, treatment has improved the overall condition of II-2 (Table 1); he restarted crawling and climbing to a standing position (+1 year, 8 months) and regained use of a walk-assist frame (+3 years, 4 months). He demonstrated improved attention and persistence at tasks (+1 year, 11 months), and from +3 years is more communicative, regaining a limited (4–6 words) vocabulary at +5 years, with improved consistency in following single-step directions. His seizure control remains effective and there has been interval improvement towards normal in background electrographic activity, albeit with persisting epileptiform discharges (Supplementary Fig. 1B). His cyclical vomiting has attenuated in frequency and duration. His nerve conduction studies confirm near total and sustained resolution of peripheral neuropathy from +3 years.

### Identification of candidate variants

WES resulted in an average coverage of the exome of 72.21 (II-1) and 88.19 (II-2), with 90.4% (II-1) and 91.8% (II-2) of the targets being covered at least 10× (Supplementary Table 1). Filtering for rare (<1% in population databases), non-synonymous variants resulted in 487 variants for II-1 and 454 variants for II-2. Overlap analysis between the two siblings did not identify variants in OMIM-reported genes known to cause neurodegeneration. Two variants were however identified in *SLC5A6*, V141Afs*34 (Chr2(GRCh37):g.27,429,780GCA>G; c.422_423delTG), and R400T (Chr2(GRCh37):g.27,426,109C>G; c.1199G>C).

Sanger sequencing of the unaffected parents showed each was a heterozygous carrier of one variant, p.V141Afs*34 (maternal) and p.R400T (paternal) (Fig. 1a), and confirmed the presence, in compound heterozygosity, of both variants in the affected individuals. The unaffected half-sibling was not tested for the paternal allele.

### Evaluation of SLC5A6 variants

Qualitative analysis of *SLC5A6* wild-type and mutant allele expression was performed by Sanger sequencing of patient cDNA. Expression of the V141Afs*34 truncated allele was decreased compared to wild-type. The R400T allele was stably expressed and was the only allele present at that location in the compound heterozygous, affected children (Fig. 1b).

Biotin uptake assays were initially performed on primary dermal fibroblasts. After growing the cells for 24 h in media supplemented with tritium-labelled biotin, cell lysates were harvested and the amount of radioactivity quantitated by scintillation counting. Whilst decreased biotin uptake was observed in cells derived from the compound heterozygous, affected children relative to their heterozygous, unaffected parents, this difference was not statistically significantly different compared to controls (Supplementary Fig. 4A).

Biotin uptake was then assessed in cultured HeLa cells transiently transfected with expression constructs for wild-type and mutant SLC5A6. Cell lysates were again harvested and quantitated after 24 h of growth in media containing radiolabelled biotin. Expression of wild-type SMVT significantly increased biotin uptake by 1.5-fold over the empty vector negative control (*p* = 0.008) (Fig. 1c). In contrast, the uptake of biotin by the R400T and V141Afs*34 mutant SMVT proteins was not different from uptake in the empty vector control (Fig. 1c). This result confirmed both variants result in a severe loss of function.

## Discussion

Here we provide consolidative evidence implicating disordered SMVT function in an infantile neurodegenerative phenotype. SMVT is a sodium-dependent transporter of biotin, pantothenate and lipoate; organic enzyme co-factors which are integral to fatty acid metabolism and cellular energy production (Fig. 1d, e).^9,10^

It is notable that prior defined inborn errors of biotin metabolism, holocarboxylase synthetase deficiency (HLCS; MIM#253270) and biotinidase deficiency (BTD; MIM#253260) share phenotypic similarities with the cases described herein. Untreated patients demonstrate progressive cognitive and neuromotor decline, often with prominent seizures, with variable response to biotin replacement therapy.^5–7,11^ Further clinical overlap is evident amongst inborn mutations affecting pantothenate metabolism, which are implicated as causative of pantothenate kinase-associated neurodegeneration (PKAN/NBIA1, PANK2; MIM#234200) and COASY protein-associated neurodegeneration (CoPAN/NBIA6, COASY; MIM#615643). In addition, neuroaxonal spheroids which were widely distributed throughout the cerebrum of patient II-1 (Supplementary Fig. 3A), are a characteristic neuropathological feature of pantothenate kinase-associated neurodegeneration.^12–14^ More recently, perturbed lipoic acid biosynthesis has been implicated in the pathological disruption of mitochondrial oxidative metabolism. Features of these disorders are varied but, in many cases, include infantile encephalopathic presentations with severe epilepsy, similar to that reported in the cases of SMVT deficiency here.^15–19^

While some degree of phenotypic heterogeneity is evident between the described sibling pair and the unrelated index case, the basis of these differences remains uncertain given the small number of cases identified to date.^4^ It is likely though, that the temporal progression of untreated disease in II-2 has contributed to more severe neurological manifestations. Further delineation of these findings will be informed by identification and longitudinal investigation of additional cases.

Despite the fact that biotin, pantothenate and lipoate feed into numerous biochemical pathways (Fig. 1d, e), biochemical analyses of metabolites in both our patients and the previously reported patient did not detect any abnormalities.^4^ This is suggestive of a redundancy in B-group vitamin transport within the gastrointestinal tract, in contrast to the critical role of SMVT in transport across the blood–brain barrier, which may explain the neurological features but lack of biochemical abnormalities.^20^ It is similarly interesting that neuroimaging features and pathological findings specific to the basal ganglia, commonly observed in disorders of biotin, pantothenate and lipoate metabolism, were not evident in our patients or the previously described individual.^4^

The mutations we identified in *SLC5A6*, V141Afs*34 (29 alleles, 0.01%) and R400T (2 alleles, 0.0008%), have both been observed at extremely low frequency in the population database gnomAD, but never in homozygosity.^21^ The V141Afs*34 variant is predicted to result in a truncated protein product, removing many of the functionally important domains (Supplementary Fig. 4B).^9,22,23^ This truncation was supported by the cDNA experiment (Fig. 1b), which also suggested nonsense-mediated decay by displaying reduced expression of this allele. The R400T variant is located on an intracellular loop of SMVT, close to a transmembrane domain (Supplementary Fig. 4B). Multiple species alignment shows the R400 residue is moderately conserved, retained in 25 of 39 vertebrate species (Supplementary Fig. 4C), with the lysine residue present in other species having similar chemical properties to arginine. The molecular basis of impaired SMVT function due to this missense mutation is unclear.

SMVT deficiency is extremely rare, with only one similar case being reported to date (Table 1).^4^ In order to identify additional patients, we contacted researchers with cohorts of patients without a genetic diagnosis for suspected mitochondrial and neurological disease, with existing WES data, as well as sharing the genotypic and phenotypic information of patient II-2 on MatchMaker Exchange.^24^ However, no additional patients with homozygous or compound heterozygous variants in *SLC5A6* were identified.

The paucity of clinically affected individuals due to pathogenic variants in *SLC5A6* may be explained by the critical role of biotin, pantothenate and lipoate in numerous metabolic pathways. The SMVT protein is solely responsible for intestinal biotin uptake and has been shown to be responsible for 89% of biotin transport across the blood–brain barrier.^9,20,25^ It is therefore likely that carrying biallelic amorphic alleles, resulting in no functional SMVT protein, is lethal. This is supported by the absence of homozygous loss-of-function variants in gnomAD. Conversely, individuals with two hypomorphic alleles may have sufficient residual SMVT function to remain clinically asymptomatic. This is similar to that observed in other metabolic disorders, such as biotinidase deficiency, where patients carrying two alleles with residual function are clinically well.^6^ Both the earlier described patient and our patients carry a single amorphic (null) allele in combination with a hypomorphic allele, likely resulting in sufficient transporter activity to support life, but inadequate to meet biological demands after birth.^4^

Complete SLC5A6 knockout mice have not been reported. Only one-third of conditional (intestine-specific) SLC5A6 knockout mice were viable and display growth retardation, decreased bone density and histological abnormalities of the gastrointestinal tract, overlapping with phenotypic features (e.g. gastrointestinal bleeding and osteopenia) present in the human patients reported to date.^4,25^ While the mechanisms underlying these varied clinical characteristics remain uncertain, it is notable that the phenotype of the conditional knockout mouse could also be rescued by over-supplementation of biotin and pantothenic acid.^26^

For patient II-2, triple replacement therapy was initiated upon molecular diagnosis of SMVT deficiency at 7 years of age. The case described in the earlier report commenced treatment at 19 months of age, which prevented further neurodegeneration and significantly improved clinical outcomes in this child.

This is the second report of biallelic *SLC5A6* mutations underpinning infantile neurodegenerative disease with subsequent therapeutic response. This result reinforces the importance of critical cellular cofactors in nervous system function and development, and emphasises the potential for significant clinical benefit, at the individual patient level, that can result from access to exploratory molecular analysis. Introduction of neonatal or even preconceptual molecular screening would allow identification of an increasing number of rare and ultra-rare novel disorders, where early intervention is supported by therapeutic benefit. In the case of patients with *SLC5A6* mutations, this provides the opportunity to dramatically attenuate or perhaps even ameliorate neurodegeneration when identified early within the disease course.

## Methods

### Patients

Written informed consent to perform WES was obtained under institutionally approved molecular diagnostic consent protocols (SA Pathology), in accordance with the Declaration of Helsinki. Experimental treatment regime was approved by the Women’s and Children’s Health Network Drug and Therapeutics Committee (30 September 2014), and access obtained following approval under the Australian Government Therapeutic Goods Administration Special Access Scheme.

### Genetic studies

WES was performed on genomic DNA samples from II-1 and II-2 at the Centre for Cancer Biology ACRF Genomics Facility. Exonic sequences were enriched using the SeqCap EZ Human Exome Library v3.0 kit (Roche NimbleGen) and libraries sequenced as 100 bp paired-end reads on the HiSeq 2000 platform (Illumina).

Read alignment to the UCSC human genome assembly hg19 was performed with the Burrows–Wheeler Aligner (v.0.6.2). Picard was used to mark duplicates and GATK (v2.7-2) was used for local realignment around indels and to recalibrate quality scores. SNV’s and small insertions and deletions were detected using GATK’s Unified Genotyper (v2.7-2).

To rule out sample identity errors, SNP calls from WES data were compared to calls from the Infinium CytoSNP-850K BeadChip assay (Illumina), performed according to the manufacturer’s protocol at the Centre for Cancer Biology ACRF Genomics Facility on genomic DNA samples.

### Variant filtering and prioritisation

WES results were first investigated for variants in OMIM genes known to be causative of neurodegeneration, but no likely pathogenic variants were identified. Results were then filtered to highlight potentially causative variants under three modes of inheritance: autosomal recessive with homozygous or compound heterozygous variants, and autosomal dominant due to parental mosaicism. Variants were only considered if they were present in both affected children, were predicted to cause a functionally important effect (SNPEFF effect moderate or high) and were rare in the general population (ExAC MAF <1% for AR or <0.1% for AD).^21^ Variants were further prioritised by only considering those predicted to be pathogenic by in-silico programmes (CADD score >20) and in conserved regions (GERP score >2). Variants present in concordant zygosity in our in-house genomic variant database were excluded (Supplementary Table 1).

Candidate variants from WES were confirmed and co-segregation assessed by Sanger sequencing of PCR-amplified genomic DNA from affected and unaffected family members.

### Gene expression analysis

Total RNA was extracted from dermal fibroblasts (I-1, I-2, II-1 and II-2) using the RNeasy Mini Kit (Qiagen). Reverse transcription was performed using the SuperScript III Reverse Transcriptase kit (Invitrogen), as per the manufacturer’s instructions, with 150 ng of Random Primers (Promega) and 1 μg of total RNA. RNA complimentary to cDNA was removed using 1 μL of RNase H (Invitrogen). Gene expression was assessed by Sanger sequencing of PCR-amplified cDNA.

### Cell culture

HeLa cell cultures were maintained in Dulbecco’s modified Eagle medium (DMEM) with 20 mM HEPES, supplemented with 10% foetal bovine serum (Sigma-Aldrich) and 1% penicillin–streptomycin–glutamine (Gibco). Cells were split every 3–4 days at a ratio of 1:2. HeLa cells were purchased from the American Type Culture Collection (ATCC, Reference CCL-2). Primary dermal fibroblast cultures were established following skin biopsy of I-1, I-2, II-1 and II-2 at the Women’s and Children’s Hospital, Adelaide. Cultured fibroblasts were maintained using basal Eagle’s medium 1 × (BME) (Gibco) with 10% foetal bovine serum, 1% l-glutamine, 200 mM and 1% penicillin–streptomycin (all from Sigma-Aldrich). Cells were split every 7–10 days at a ratio of 1:2.

### Generation of expression constructs

Wild-type SLC5A6 expression clones were obtained from OriGene (RC204865). Mutant alleles were created by site-directed mutagenesis, following the manufacturer’s protocol for the Quikchange II XL Site-Directed Mutagenesis Kit (Stratagene). Products were incubated with the *DpnI*restriction enzyme to digest the parental (non-mutated) plasmid dsDNA. Wild-type, mutagenized and pCMV6-entry empty vector clones were transformed into XL-10 Gold Ultracompetent Cells (Agilent Technologies) and incubated overnight. Single colonies were grown overnight in liquid culture and clones purified using the QIAfilter Plasmid Midi Kit (QIAGEN). The entire *SLC5A6* insert in wild-type and mutant clones was verified by Sanger sequencing in both directions.

### Transfection of HeLa cells

HeLa cells were transfected using the Lipofectamine 3000 reagent (Invitrogen) according to the manufacturer’s protocol. Cells were seeded in 12-well plates (2 × 10^5^ cells) and incubated overnight. The following day, cells were transfected using 0.25 and 1 μg, respectively, of empty-vector, wild-type or mutant expression construct.

### Biotin uptake assay

Biotin uptake studies were performed using primary dermal fibroblasts (I-1, I-2, II-1 and II-2), and HeLa cells transfected with empty-vector pCMV6 or constructs expressing wild-type SLC5A6 or mutant protein.^27^ Dermal fibroblasts were seeded in 12-well plates (2.5 × 10^5^ cells) in basal Eagle’s medium 1 × (BME) (Gibco) with 20% foetal bovine serum (Sigma-Aldrich) and 1% l-glutamine. 24 h after seeding, cells were incubated in Krebs–Ringer buffer (133 mM NaCl, 4.93 mM KCl, 1.23 mM MgSO_4_, 0.85 mM CaCl_2_, 5 mM glucose, 5 mM glutamine, 10 mM HEPES and 10 mM MES, pH 7.4) containing 20 nM ^3^H biotin (American Radiolabeled Chemicals). Biotin uptake was measured after 24 h by washing cells twice with 1 ml of ice-cold Krebs–Ringer buffer then lysed in 0.2 ml of 1 M NaOH. Radioactive content in the cell lysates was quantitated using a scintillation counter with OptiPhase Supermix scintillation cocktail (Perkin Elmer). Protein content in the lysates was measured using a Bradford protein assay kit. Uptake assays in transfected HeLa cells were performed as above except cells were incubated in Krebs–Ringer buffer containing 80 nM ^3^H biotin for 24 h post-transfection. Each transfection and uptake assay were performed in triplicate, and the results calculated as cpm/mg of lysate. The data from four discrete assays (*n* = 4) was combined for statistical analysis. A two-tailed Student’s *t*-test was performed comparing the empty vector and expression constructs.

### Reporting summary

Further information on experimental design is available in the Nature Research Reporting Summary linked to this paper.

## Supplementary information

Supplementary Information

Reporting Summary Checklist

## Data Availability

Sequence data has been deposited at the European Genome-phenome archive, which is hosted by the European Bioinformatics Institute, under accession #EGAS00001003861. All unique materials and datasets generated and/or analysed during the current study are available from the corresponding author on reasonable request.
