# Supplementary material for: Identification and targeted management of a neurodegenerative disorder caused by biallelic mutations in SLC5A6
Source: NPJ Genom Med. 2019 Nov 14;4:28. doi: 10.1038/s41525-019-0103-x (PMC6856110; doi:10.1038/s41525-019-0103-x)
Supplement: Supplementary file 1 — Supplementary Information [file 41525_2019_103_MOESM1_ESM.pdf]

Supplementary Information

for

IDENTIFICATION AND TARGETED MANAGEMENT OF A NEURODEGENERATIVE DISORDER CAUSED BY BIALLELIC MUTATIONS IN SLC5A6

Alicia B Byrne[1,8], Peer Arts[1], Steven W Polyak[2,8], Jinghua Feng[3,8], Andreas W Schreiber[2,3,8], Karin S Kassahn[2,4], Christopher N Hahn[1,4,8,9], Dylan A Mordaunt[5], Janice M Fletcher[4], Jillian Lipsett[6], Drago Bratkovic[5,9], Grant W Booker[2], Nicholas J Smith[7,9,*,^], Hamish S Scott[1,3,4,8,9,*,^]

[1]Genetics and Molecular Pathology Research Laboratory, Centre for Cancer Biology, An alliance between SA Pathology and the University of South Australia, Adelaide, SA, Australia

[2]School of Biological Sciences, University of Adelaide, Adelaide, SA, Australia

[3]ACRF Cancer Genomics Facility, Centre for Cancer Biology, An alliance between SA Pathology and the University of South Australia, Adelaide, SA, Australia

[4]Department of Genetics and Molecular Pathology, SA Pathology, Adelaide, SA, Australia

[5]South Australian Clinical Genetics Service, Women's and Children's Hospital, North Adelaide, SA, Australia

[6]Department of Surgical Pathology, SA Pathology, North Adelaide, SA, Australia

[7]Department of Neurology, Women's and Children's Hospital, North Adelaide, SA, Australia

[8]School of Pharmacy and Medical Sciences, University of South Australia, Adelaide, SA, Australia

[9]School of Medicine, University of Adelaide, Adelaide, SA, Australia

*These authors contributed equally to this work

^Corresponding authors (nicholas.smith@sa.gov.au, hamish.scott@sa.gov.au)

SUPPLEMENTARY INFORMATION

Supplementary Notes

Supplementary Figures

Supplementary Tables

SUPPLEMENTARY NOTES

Clinical Description

The family consists of a female (II-1) and male (II-2) sibling pair, born to unaffected, unrelated parents (Figure 1A), each manifesting profound neurodevelopmental delay during infancy; both patients were born at term gestation, without complication, following a benign antenatal period. Birth occipitofrontal head circumference (OFC) was 34cm (50th centile) on II-1 and 34.5cm (50th centile) in II-2; postnatal reduction in OFC proved evident in both children, with an OFC at death of 46cm (2nd-10th centile) in II-1 at 2 years, 7 months of age and 51cm (2nd-10th centile) in II-2 at current assessment (age 10 years, 5 months of age).

Infantile neurodevelopmental was age appropriate, with a plateau in global development from 14 months and 12 months in II-1 and II-2 respectively: II-1 achieved independent walking at 15 months of age, however progressively ataxic gait ensued with regression to crawling from 20 months and non-ambulation at 24 months. She achieved a vocabulary of 15-20 words by 15 months with a retraction to 10 words at 23 months and purposeful use of mum and dad only, by 30 months. II-2 commenced independent steps at 12 months, although never walked over distance; he utilised a walking assist device to the age of 5 years, 6 months, at which time he regressed to crawling and latterly mobilising by ‘commando crawl’. He achieved a vocabulary of 10 single words by 15 months of age, regressing to a non-verbal state by 24 months.

Neurological assessment demonstrated progressive truncal ataxia with dyskinetic appendicular movements from 14 months (II-1) and 12 months (II-2); myotatic reflexes proved moderately brisk, without pathological clonus and extensor plantar responses were evident in II-1 only. Notably, lower limb myotatic reflexes temporally diminished from 3 years of age in II-2, secondary to evolution of a progressive mixed axonal and demyelinating sensorimotor peripheral neuropathy. Overall, axial and appendicular tone was reduced. Bulbar dysfunction with secondary aspiration, precipitated nasogastric tube insertion in II-1 at 2 years 7 months of age and gastrostomy at 6 years, 3 months in

II-2. Hypermetropia was present in both children with functional visual acuity intact; a unilateral, left sided esotropia was onset from 14 months in II-1, while II-2 developed binocular esotropia from around 12 months, with dyskinetic eye movements and jerk nystagmus in both the horizontal and vertical planes. Fundoscopic examination revealed mildly pallid discs for both siblings. Auditory acuity proved grossly intact in both children; hyperacusis with a prominent startle response was noted, attenuating latterly in II-2.

II-2 developed mixed semiology seizures including focal dyscognitive, absence, tonic spasms and generalised convulsive seizures with electrographic findings of encephalopathy (background slowing), with generalised and independent multifocal spike-wave discharges (Supplementary Figure 1A). Seizures proved well controlled on a regimen of levetiracetam, topiramate and vigabatrin. Treatment refractory cyclical vomiting has been an additional feature in II-2, from 3 years, 5 months of age. Both siblings manifest mild atopy and from late infancy, hypoproteinaemia of undefined cause (presumed nutritional) was evident. Curiously, II-2 developed digital clubbing, from around 3 years of age - in the absence of predisposing systemic pathology. He was also managed for tracheobronchomalacia and gastro-esophageal reflux which did not manifest in his sister (II-1).

Neuroimaging proved non-specific. II-1 demonstrated T2-weighted signal hyperintensity involving the periventricular and parieto-occipital white matter (Supplementary Figure 2A) and a small haemorrhagic focus in the right cerebellar hemisphere (likely sustained perinatally), at 1 year, 8 months of age. II-2, at 7 years, 5 months of age, demonstrated progressive cerebello-pontine atrophy on serial imaging with cerebral white matter volume loss and a thinned corpus callosum; T-2 weighted images demonstrated non-restricting signal hyperintensity within the central tegmental tracts and peritrigonal regions bilaterally (Supplementary Figure 2B and 2C). Single voxel proton-spectroscopy (1H-MRS) at 31 and 144 milliseconds revealed a reduction in the neuronal marker N-acetylaspartate (NAA) and broad lactate doublets suggestive of anaerobic glycolysis within the basal ganglia and fronto-parietal white matter. It is notable that isolated radiographic features of basal

ganglia involvement, commonly witnessed amongst acquired and inborn disorders of biotin, pantothenate and lipoate metabolism, were not evident in either sibling.

Neurophysiological assessment, neuroimaging, biochemical, single-gene testing and mitochondrial genome sequencing did not provide a diagnosis for this family. Respiratory chain enzyme activity, performed on skeletal muscle in II-1, demonstrated an isolated reduction in complex 1 (8 nmol/min/mg [RR19-72]), however hepatic activity proved normal. Final diagnosis proved reliant on full exome analysis.

II-1 died at 2 years 7 months of age, secondary to acute gastrointestinal haemorrhage, following perforation of a duodenal ulcer. In addition, post-mortem examination confirmed relative cardiomegaly and hepatic congestion, suggestive of right heart failure; pulmonary features of aspiration pneumonia were also noted. II-2 has not manifested clinical features of cardiomegaly to date, and a screening echocardiogram at 8 years, 9 months of age proved within normal limits; non-specific ST-segment and T-wave changes, without electrographic features of ventricular hypertrophy were noted on ECG. Neuropathological autopsy (II-1) confirmed a macroscopically small cerebrum and cerebellum for age (brain weight 977g), with preservation of the overall neural architecture. Histopathological analysis demonstrated a global increase in neuronal and glial density, with reactive gliotic change. Numerous axonal spheroids (Supplementary Figure 3A), measuring up to 100µm in diameter, were ubiquitously present, throughout the neuraxis, with the highest density within the posterior medulla (including the gracile and cuneate nuclei). Ultrastructural appearances demonstrated aggregations of mitochondria and ribosomes with filamentous (8nm diameter) and tubular structures (25nm diameter) also present. Peripheral nerve sampling confirmed non-specific regions of focal thickening, without the presence of true spheroids. Features of denervation atrophy were evident on skeletal muscle sampling. Of interest, ultrastructural analysis of a cutaneous biopsy in II-2 did not reveal morphological changes in the cutaneous nerve fibres, though did demonstrate cytoplasmic membrane-bound inclusions in both fibroblasts and Schwann cells (Supplementary

Figure 3B), suggestive of disordered endosomal-lysosomal function. Screening lysosomal hydrolase activities were normal.

Upon molecular diagnosis, II-2 was commenced upon triple replacement therapy at 7 years, 1 month (Table 1), comprising Biotin, Pantothenate and α-lipoic acid – administered parenterally in the context of his cyclical vomiting and under rationale of high gastrointestinal expression of the dysfunctional transporter. To date, treatment response has proved encouraging; subjective improvements in patient II-2's social interaction and stamina were evident from +3 months (post initiation of therapy) and have continued. II-2 regained the ability to crawl and climb to a standing position at +1 year, 8 months and is utilising a walk assist frame without difficulty from +3 years 4 months. Cognitive improvements have been documented; at +1 year, 11 months the child demonstrated increasing social interaction with improved attention and persistence at tasks. He will utilise a cup to drink and feed himself from a spoon. From +3 years began using 'mum' purposefully and at +5 years has regained a limited (4-6 word) vocabulary and demonstrates improved consistency in following simple (single-step) directions. While his binocular esotropia persists, this too has improved and his nystagmus has near completely resolved. His seizure control remains effective (with a reduction in his anticonvulsant requirements) and there has been an interval improvement towards normal, in his background electrographic activity, albeit with persisting epileptiform discharges (Supplementary Figure 1B). His cyclical vomiting has attenuated in frequency and duration. As noted, his nerve conduction studies have confirmed near total resolution of his peripheral neuropathy, with normalisation of electrographic features at +3 years.

SUPPLEMENTARY FIGURES

a

b

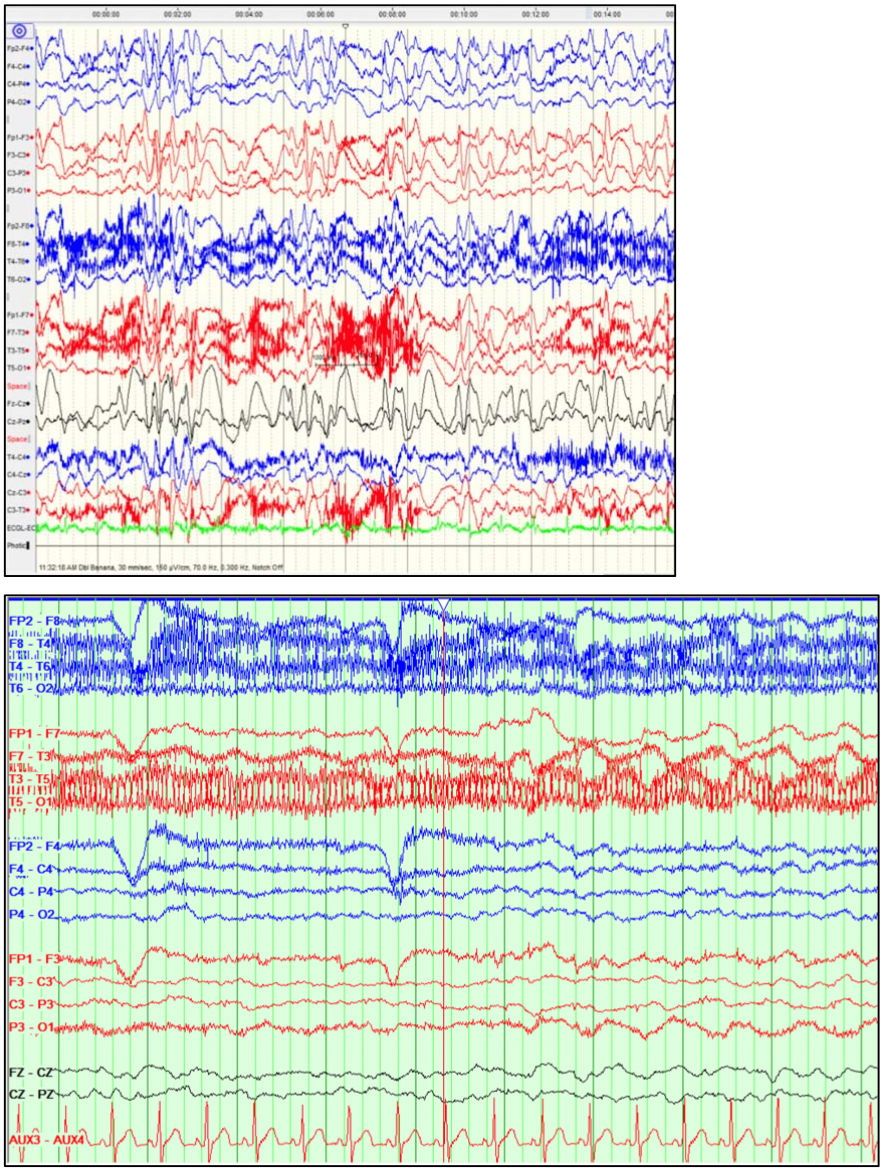

Supplementary Figure 1: Pre- and post- treatment electroencephalogram results for II-2. a) Electroencephalogram [II-2: Pre-treatment; 5y 5m age]: Background demonstrates predominant 2-4Hz rhythms with frequent independent and bisynchronous spike-slow wave and poly-spike epileptiform discharges. Moderate EMG artefact present. [NicoletOne^{TM} 32 Channel: International 10-20 electrode placement; Double Bananna montage presented]. **b)** Electroencephalogram [II-2: Post-treatment; +4y, 8m]: Background demonstrates predominant 4-6Hz rhythms with admixed 3-4 Hz activity. Moderate EMG artefact present. [Xltek c : International 10-20 electrode placement; Double Bananna montage presented].

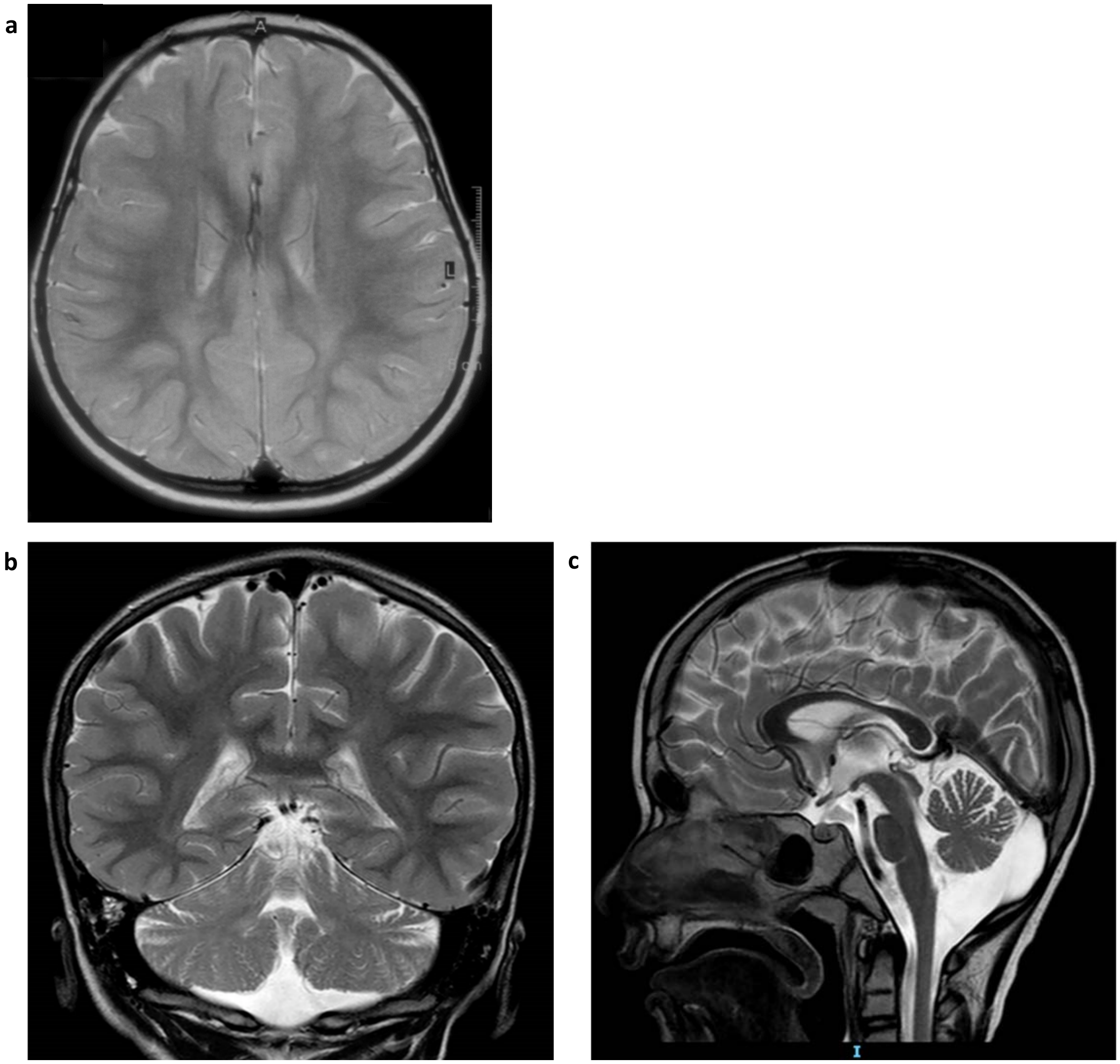

Supplementary Figure 2: MRI findings of II-1 and II-2. a) II-1 (1y, 8m): Axial, T2-weighted image; demonstrates signal hyperintensity involving the periventricular and parieto-occipital white matter [Phillips Intera^{TM} 1.5T]. **b)** and **c)** II-2 (7y, 5m; pre-treatment): Coronal (b) and saggital (c), T2-weighted images; demonstrating mild cerebral, cerebellar and brainstem atrophy. A thin corpus callosum is evident and signal hyperintensity present within the peritrigonal regions [Phillips Ingenia^{TM} 1.5T].

a
b
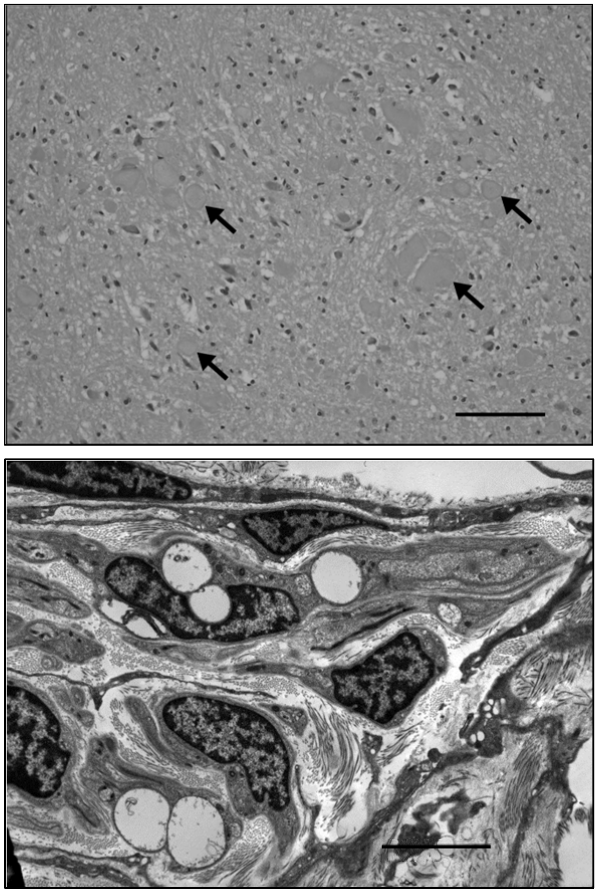

Supplementary Figure 3: Histology findings in brain and peripheral nerve of II-1 and II-2, respectively. **a)** Photomicrograph showing background gliosis with the presence of numerous variably sized neuroaxonal spheroids (arrows depict selected examples); II-1, autopsy (scale: 50 µm). **b)** Photomicrograph of peripheral nerve showing Schwann cells with membrane-bound inclusions; II-2, cutaneous biopsy (scale: 5 µm).

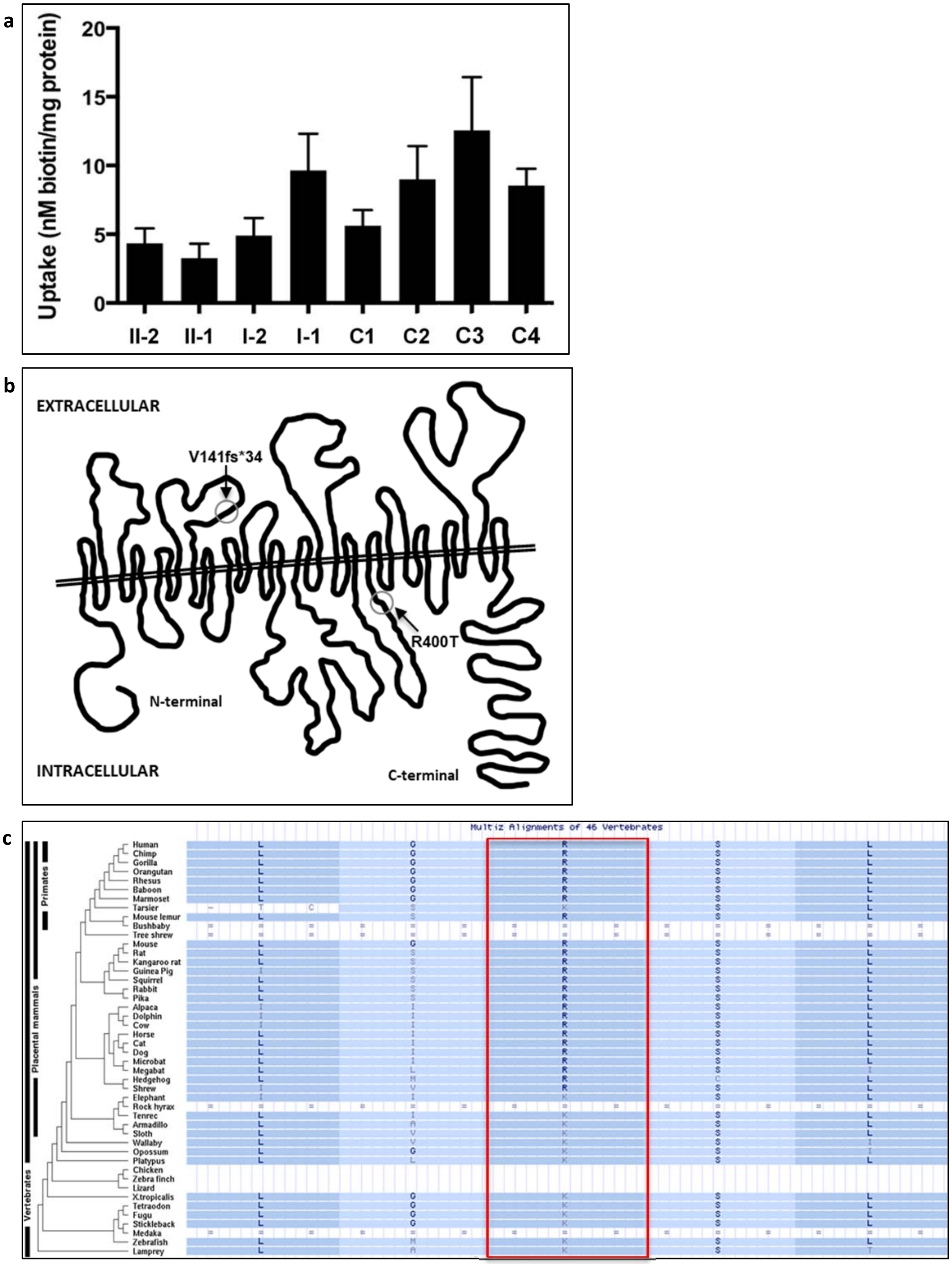
a
Uptake (nM biotin/mg protein)
0
5
10
15
20
II-2
II-1
I-2
I-1
C1
C2
C3
C4
b
EXTRACELLULAR
V141fs*34
R400T
N-terminal
INTRACELLULAR
C-terminal
c
Multiz Alignments of 46 Vertebrates
Primates
Placental mammals
Vertebrates
Human
Chimp
Gorilla
Orangutan
Rhesus
Baboon
Marmoset
Tarsier
Mouse lemur
Bushbaby
Tree shrew
Mouse
Rat
Kangaroo rat
Guinea Pig
Squirrel
Rabbit
Pika
Alpaca
Dolphin
Cow
Horse
Cat
Dog
Microbat
Megabat
Hedgehog
Shrew
Elephant
Rock hyrax
Tenrec
Armadillo
Sloth
Wallaby
Opossum
Platypus
Chicken
Zebra finch
Lizard
X.tropicalis
Tetraodon
Fugu
Stickleback
Medaka
Zebrafish
Lamprey

Supplementary Figure 4: Characterisation of V141Afs*34 and R400T variants in SLC5A6 and their functional effect. **a)** Uptake of radiolabelled biotin by primary dermal fibroblasts from family members and controls. Uptake in the affected children (II-1 and II-2) is decreased compared to their unaffected parents (I-1 and I-2), but not statistically significantly decreased compared to controls (C1-C4). Data show the mean and standard error of the mean (*n*=4). **b)** Predicted topology of the SMVT protein in the plasma membrane (adapted from Ghosal & Said, 2011).[9] Amino acids mutated in this family circled in grey. **c)** Multiple-species alignment of the region of SLC5A6 protein sequence containing the R400T variant, conserved in 25/39 species.

SUPPLEMENTARY TABLES

Supplementary Table 1: Exome sequencing results and variant filtering outcomes

**Sample ID**	**II-1**	**II-2**
Total mapped data (Gb)	6.24	5.11
Mean coverage	97.24	79.63
Median coverage	73	63
% bases covered >10x	91.6	90.2
% bases covered >20x	88.2	86.1

Total variants (passed VQSR, depth >10x)	207,589	195,788
Coding, non-synonymous variants	16,209	15,824
Rare <1% (ExAC, in-house)	487	454
VAF >35%	12	8
Overlapping recessive - comp het	6 variants (3 genes)<br>SLC5A6 (Val141Alafs*34, Arg400Thr)<br>GCOM1 (Gln254Pro, Glu337Gly)<br>ERN1 (Leu352dup, Tyr354Thrfs*2)	
Functional association (UniProtKB): Biotin, lipoate and pantothenate transport	SLC5A6	
