# Supplementary material for: Identification and targeted management of a neurodegenerative disorder caused by biallelic mutations in SLC5A6
Source: NPJ Genom Med. 2019 Nov 14;4:28. doi: 10.1038/s41525-019-0103-x (PMC6856110; doi:10.1038/s41525-019-0103-x)
Supplement: Supplementary file 2 — Reporting Summary Checklist [file 41525_2019_103_MOESM2_ESM.pdf]

nature research

Corresponding author(s): Hamish Scott

Last updated by author(s): Sep 24, 2019

Reporting Summary

Nature Research wishes to improve the reproducibility of the work that we publish. This form provides structure for consistency and transparency in reporting. For further information on Nature Research policies, see Authors & Referees and the Editorial Policy Checklist.

Statistics

For all statistical analyses, confirm that the following items are present in the figure legend, table legend, main text, or Methods section.

n/a	Confirmed	
☐	☒	The exact sample size (n) for each experimental group/condition, given as a discrete number and unit of measurement
☐	☒	A statement on whether measurements were taken from distinct samples or whether the same sample was measured repeatedly
☐	☒	The statistical test(s) used AND whether they are one- or two-sided<br>*Only common tests should be described solely by name; describe more complex techniques in the Methods section.*
☒	☐	A description of all covariates tested
☒	☐	A description of any assumptions or corrections, such as tests of normality and adjustment for multiple comparisons
☐	☒	A full description of the statistical parameters including central tendency (e.g. means) or other basic estimates (e.g. regression coefficient) AND variation (e.g. standard deviation) or associated estimates of uncertainty (e.g. confidence intervals)
☐	☒	For null hypothesis testing, the test statistic (e.g. F, t, r) with confidence intervals, effect sizes, degrees of freedom and P value noted<br>*Give P values as exact values whenever suitable.*
☒	☐	For Bayesian analysis, information on the choice of priors and Markov chain Monte Carlo settings
☒	☐	For hierarchical and complex designs, identification of the appropriate level for tests and full reporting of outcomes
☒	☐	Estimates of effect sizes (e.g. Cohen's d, Pearson's r), indicating how they were calculated

Our web collection on statistics for biologists contains articles on many of the points above.

Software and code

Policy information about availability of computer code

Data collection	No software was used.
Data analysis	Burrows-Wheeler Aligner (v.0.6.2) - mapping. GATK (v2.7-2) - variant calling. Variant Effect Predictor - variant annotation.

For manuscripts utilizing custom algorithms or software that are central to the research but not yet described in published literature, software must be made available to editors/reviewers. We strongly encourage code deposition in a community repository (e.g. GitHub). See the Nature Research guidelines for submitting code & software for further information.

Data

Policy information about availability of data

All manuscripts must include a data availability statement. This statement should provide the following information, where applicable:

- Accession codes, unique identifiers, or web links for publicly available datasets
- A list of figures that have associated raw data
- A description of any restrictions on data availability

Sequence data has been deposited at the European Genome-phenome archive, which is hosted by the European Bioinformatics Institute, under accession #EGAS00001003861. All unique materials and datasets generated and/or analysed during the current study are available from the corresponding author on reasonable request.

Field-specific reporting

Please select the one below that is the best fit for your research. If you are not sure, read the appropriate sections before making your selection.

☒ Life sciences ☐ Behavioural & social sciences ☐ Ecological, evolutionary & environmental sciences

For a reference copy of the document with all sections, see nature.com/documents/nr-reporting-summary-flat.pdf

Life sciences study design

All studies must disclose on these points even when the disclosure is negative.

Sample size	No sample-size calculations were performed. Sample size was determined to be adequate based on consistency of measurable differences between groups.
Data exclusions	No data were excluded from the analyses.
Replication	Assays were performed in triplicate and data from four discrete assays (n=4) was combined for statistical analysis. Replicate experiments were successful.
Randomization	Samples were not randomized - not relevant to study determining molecular basis of disease.
Blinding	Investigators were not blinded. Data reported for uptake assays are quantitative not subjective.

Reporting for specific materials, systems and methods

We require information from authors about some types of materials, experimental systems and methods used in many studies. Here, indicate whether each material, system or method listed is relevant to your study. If you are not sure if a list item applies to your research, read the appropriate section before selecting a response.

Materials & experimental systems

n/a	Involved in the study	
☒	☐	Antibodies
☐	☒	Eukaryotic cell lines
☒	☐	Palaeontology
☒	☐	Animals and other organisms
☐	☒	Human research participants
☒	☐	Clinical data

Methods

n/a	Involved in the study	
☒	☐	ChIP-seq
☒	☐	Flow cytometry
☒	☐	MRI-based neuroimaging

Eukaryotic cell lines

Policy information about cell lines

Cell line source(s)	ATCC , Reference Number CCL-2
Authentication	Cell lines were originally purchased from ATCC. Before use, cells were sent to CellBank Australia for gDNA analysis and were shown to be 96% identical to HeLa (ATCC: CCL-2). Additionally only low passage cells (passage number <10) were used for experiments.
Mycoplasma contamination	Cells were confirmed negative for mycoplamsa.
Commonly misidentified lines (See ICLAC register)	No cell lines used are listed in the database of commonly misidentified cell lines.

Human research participants

Policy information about studies involving human research participants

Population characteristics	Blood and fibroblasts from male (6) and female (2) sibling pair, with profound neurodevelopmental delay during infancy, and their unaffected, unrelated parents.
Recruitment	The affected sibling pair was recruited by clinical care provider (Women's and Children's hospital Adelaide, Australia) since no genetic abnormalities were detected through standard diagnostic testing.

Ethics oversight	Women's and Children's Health Network and SA Pathology

Note that full information on the approval of the study protocol must also be provided in the manuscript.
